# The Impact of Resilience on Mental Health Outcomes in Dyads of Informal Caregivers and Their Older Care Recipients

**DOI:** 10.1093/geroni/igaf122.2939

**Published:** 2025-12-31

**Authors:** Jingyuan Liu

**Affiliations:** The Hong Kong Polytechnic University, Hong Kong, China (People’s Republic)

## Abstract

**Background:**

Resilience is widely recognized as a critical protective factor for mental health. However, existing research has predominantly focused on its individual-level effects, overlooking potential dyadic mechanisms in highly interdependent relationships. This gap is particularly salient in caregiver-older care recipient dyads, where mutual psychological interdependence is pronounced. To address this limitation, the present study applies the Actor-Partner Interdependence Model (APIM) to examine how resilience operates within caregiver-recipient dyads.

**Methods:**

A sample of 120 dyads of caregiver (Mage = 60.31 years, SDage = 11.10 years) and older care-recipients (Mage = 76.06 years, SDage = 8.46 years) were recruited in China. The APIM approach was used to analyze the dyadic effects of resilience on mental health outcomes, including depression, anxiety, and general well-being. Multilevel modeling (MLM) was employed to estimate the resilience’s actor and partner effects on mental health outcomes within the dyads.

**Results:**

Results from APIM revealed the actor effect of resilience on both caregivers and care-recipients’ own mental health outcomes. A significant partner effect on anxiety was observed (β = −0.133, p < 0.05), indicating that a resilient partner can significantly reduce one’s own anxiety levels.

**Conclusion:**

The findings reveal that resilience influences not only an individual’s mental health but also their partner’s anxiety. This underscores the need to consider reciprocal resilience dynamics in resilience building programs. Interventions targeting only one member of the dyad may miss the crucial interdependencies between caregivers and recipients. Therefore, integrated interventions that enhance resilience in both parties should be prioritized in future service design.

